# Small-Sized Circular Genomes Similar to Genome of Porcine Circovirus 2

**DOI:** 10.1128/genomeA.00093-13

**Published:** 2013-03-28

**Authors:** Wei Luo, Dun Zhao, Xing-Long Yu, Meng Ge, Run-Cheng Li, Da-Liang Jiang

**Affiliations:** College of Veterinary Medicine, Hunan Agricultural University, Changsha, China

## Abstract

Circular genomes smaller than and similar to the genome of porcine circovirus 2 were obtained from pig tissues along with the full-length genome of porcine circovirus 2. The 922-, 839-, and 617-nucleotide-long genomes exhibit high homology to the *rep* gene plus the origin of replication sequence of porcine circovirus 2.

## GENOME ANNOUNCEMENT

Porcine circovirus 2 (PCV2) is a kind of circular single-stranded DNA virus that belongs to the *Circovirus* genus in the *Circoviridae* family (1). PCV2 is involved in many disease syndromes of pigs, and the genome of PCV2 is 1,767 or 1,768 nucleotides (nt) in length (2, 3). PCV2 could be divided into three genotypes, namely, PCV2a/genotype 2/group 2, PCV2b/genotype 1/group 1, and PCV2c/genotype 3/group 3 (4). PCV2b/genotype 1/group 1 is the strain predominating the pig populations (5, 6) and can be divided into subgroups 1A, 1B, and 1C (7).

Rearranged genomes that harbor the origin of the replication (*ori*) sequence of PCV2 and whose coding regions were mainly similar to the *cap* gene of PCV2 were reported (8–12). Here we obtained novel PCV2-like isolates HN2-3, HN6-1, and HN3-1 from tissues of sick pigs collected in 2008 from locations in Hunan Province, China. Genomes of these isolates were obtained by reverse PCR followed by T-A cloning using vector pMD18-T. The recombinant plasmids were sequenced with an ABI 3730 XL genome sequencer (Sangon Company, Shanghai, China). Sequences were analyzed using DNAStar.

The circular genomes of isolates HN2-3, HN6-1, and HN3-1, which were recovered from different pigs which also harbored the full-length PCV2 genome, are 922, 839 and 617 nt in length, respectively. The genomes of isolates HN2-3 and HN6-1 contain the *ori* sequence of PCV2, characterized by a stem-loop structure with a nonamer motif (AAGTATTAC) at the apex of the stem-loop and 3 repeats of hexamer motifs (CGGCAG) following the right leg of the stem-loop, which serve as the binding sites for the replicases. To our surprise, the genome of isolate HN3-1 contains only part of the *ori* sequence covering the right leg of the stem-loop (GCGCACTT) and the 3 repeats of the hexamer motifs, and it is intriguing if this partial *ori* still has the replication function.

Placing the *ori* sequence ACCAGCGCACTT (for HN2-3 and HN6-1) or GCGCACTT (for HN3-1) at the start of the genome sequences, the genome sequence of HN3-1 from 51 to 694 nt displays high homology to the *rep* gene of PCV2, and that from 695 to 889 nt shows high homology to the *cap* gene of PCV2. The genome sequence of HN6-1 from 51 to 532 nt is highly similar to the *rep* gene of PCV2, and from 750 to 806 nt, it is highly similar to the *cap* gene of PCV2. The 217-nt sequence between 532 and 750 nt on the genome of HN6-1 is quite different from PCV2 but exhibits 65% homology with a sequence on *Homo sapiens* chromosome 5, making the genome sequence of HN6-1 more interesting. The genome sequence of isolate HN3-1 from 47 to 617 nt shows high homology only to the *rep* gene of PCV2. Therefore, the described genomes include the open reading frame encoding the truncated Rep protein of PCV2 or a protein somewhat different from the PCV2 Rep protein.

### Nucleotide sequence accession numbers.

The complete genome sequences of isolates HN2-3, HN6-1, and HN3-1 were submitted to the GenBank data library under the accession numbers KC415247, KC415249, and KC415248.
